# A joint photoacoustic imaging and broadband spectral analysis for early-stage intraoperative pathology assessment: A case study with colorectal cancer

**DOI:** 10.1016/j.pacs.2025.100712

**Published:** 2025-03-04

**Authors:** Fan Yang, Zhengduo Yang, Zheng Zhu, Siwei Zhu, Wei Song, Yong Yang, Xiaocong Yuan

**Affiliations:** a Research Center for Frontier Fundamental Studies, Zhejiang Laboratory, Hangzhou 311100, China; b Nanophotonics Research Center, Shenzhen Key Laboratory of Micro-Scale Optical Information Technology, Institute of Microscale Optoelectronics, Shenzhen University, Shenzhen 518060, China; c Department of Pathology, Tianjin Union Medical Center, Tianjin 300121, China; d The Institute of Translational Medicine, Tianjin Union Medical Center of Nankai University, Tianjin 300121, China

**Keywords:** Photoacoustic imaging, Photoacoustic spectral analysis, Optical surface wave, Intraoperative, Micro-infiltration

## Abstract

Accurate and rapid intraoperative diagnosis of micro-infiltration in early-stage tumors presents a formidable challenge for decades. Here, we propose a novel diagnostic approach, that combines Photoacoustic Morphological Imaging (PAMI) with an *in situ* broadband Photoacoustic Spectral Analysis (PASA), to implement intraoperative assessment of early-stage tumor while its high-frequencies between 50 and 150 MHz respond to various nuclei specifically. Our system, a broadband Ultraviolet Photoacoustic Microscopy (bUV-PAM), uniquely integrates ultraviolet laser-induced nucleus-specific photoacoustic excitation with broadband photoacoustic detection (up to 176 MHz at −6 dB) via an optical surface wave sensor. This approach facilitates the simultaneous acquisition of morphological and spectral information from unstained tissue sections, yielding a comprehensive dual-modality virtual slice within a single raster scan. Using human colorectal tissue samples, we applied the joint PAMI and *in situ* PASA approach across 6 case groups. Morphological features in PAMI showed a high concordance with Hematoxylin and Eosin (H&E) staining, whereas micro-infiltrative features were too indistinct to be identified in both PAMI and H&E images. In contrast, the PASA effectively distinguishes between micro-infiltrated and non-infiltrated tissues, a finding validated by subsequent Immunohistochemical (IHC) assessments. The preliminary results suggest that the joint approach holds potential to enhance intraoperative detection of micro-infiltration, thereby offering a promising avenue for accurate and rapid surgical margin assessment.

## 1 Introduction

Malignant tumors pose a significant threat to human health and life expectancy, with surgical resection remaining a cornerstone of cancer treatment due to the limitations of numerous oncologic drugs. Therefore, accurate intraoperative diagnosis is crucial for enhancing surgical outcomes and patient survival [1]. Clinically, intraoperative pathology relies primarily on Hematoxylin and Eosin (H&E) staining of frozen sections to evaluate tissue architecture and cellular morphology, but it often misses subtle morphological signs of certain early-stage tumors [1], [2], [3]. These pathological details are more reliably discerned through the molecular insights provided by Immunohistochemical (IHC) staining [2], [3]. Nevertheless, the time-consuming preparation required for IHC, often extending over days, significantly limits its intraoperative application. Although some IHC protocols have been developed specifically for intraoperative use, their nascent stage necessitates further validation [4], [5]. Recent advances in deep learning have attempted to convert H&E staining images into their IHC counterparts, yet the uncertain correlation between H&E and IHC images limits the clinical efficacy in making accurate IHC predictions [6], [7]. Consequently, the quest for a novel method that can directly quantify early-stage tumor characteristics for rapid intraoperative pathological diagnosis has remained a long-standing challenge.

Photoacoustic Morphological Imaging (PAMI) [8], [9], [10], [11] emerges as a promising solution for rapid intraoperative pathological diagnosis [12], [13], [14], [15]. Ultraviolet Photoacoustic Microscopy (UV-PAM) exploits the strong optical absorption of nuclei at a wavelength of 266 nm to facilitate high-contrast, specific nucleus imaging without the need for exogenous contrast agents [12]. Optical-resolution UV-PAM leverages focused laser beams for photoacoustic excitation to achieve diffraction-limited transverse resolution down to the subcellular level [13]. Validated by numerous studies, the results of UV-PAM are comparable to those of H&E staining, positioning it as an excellent candidate for rapid intraoperative pathological diagnosis due to its combined advantages of rapid imaging acquisition, label-free imaging, and high resolution [12], [13], [14], [15]. Various UV-PAM schemes have been developed for rapid intraoperative pathologic diagnosis applications, such as assessing surgical margins in fresh human breast [14] and non-decalcified bone tissue [15]. Despite these advancements, current UV-PAM techniques primarily serve as an alternative to H&E staining for capturing morphological features, often inadequately characterizing micro-infiltrated tumors due to their limited ability to discern intrinsic properties of the tissues.

Photoacoustic Spectral Analysis (PASA) [16], [17], [18], [19], [20], [21], [22], [23], [24], that is the photoacoustic power spectral analysis, is an emerging technology capable of detailed characterization of biological particle, including the assessment of red blood cell aggregation [20], determination of blood oxygen levels [20], microbial cell identification [21], and tumor type differentiation [22], [23]. Previous research has revealed a robust correlation between the photoacoustic spectral characteristics of biological particles and their properties such as acoustic velocity, diameter, and shape [24]. The progression from non-infiltrated to micro-infiltrated tumors in biological tissue, characterized by an increase in nuclear genetic material (DNA), alters the nuclei internal properties, resulting in detectable variations in the photoacoustic frequency response [25], [26], [27]. In this work, we propose a PASA approach for the identification of micro-infiltrated tumors that uses focused ultraviolet laser to selectively excite photoacoustic signals from cell nuclei, reducing the effect of nuclear morphology and spatial distribution on the photoacoustic spectra and facilitating the detection of nuclear properties associated with early-stage cancer [17]. Unlike traditional morphological photoacoustic or H&E staining images, PASA provides a quantitative assessment of the nuclear properties, facilitating the differentiation of micro-infiltration in biological tissues, thus providing additional diagnostic insights aligned with IHC without the need for extended processing.

In this paper, we present, for the first time, an intraoperative technique for rapid identification of micro-infiltrated tumors by integrating PAMI with *in situ* PASA. The proposed approach employs broadband Ultraviolet Photoacoustic Microscopy (bUV-PAM), which integrates a Total Internal Reflection (TIR) sensor for broadband photoacoustic signal detection [28], [29] and a focused ultraviolet laser for targeted nuclear photoacoustic excitation, aiming at creating a dual-modality virtual slice that enables simultaneous histomorphologic visualization and quantitative nuclear property analysis within 20 min [29]. Using human colorectal tissue samples, PAMI demonstrates strong agreement with traditional histopathological assessments from H&E staining. Moreover, PASA demonstrates high specificity in characterizing the properties of cell nuclei, effectively discriminating between normal, adenoma, and adenocarcinoma colorectal tissues. Through joint PAMI and *in situ* PASA approach across 6 groups of human colorectal tissues, we have identified a clear distinction between non-infiltrative and micro-infiltrative tissues, a differentiation further validated by IHC. Thus, the joint PAMI and *in situ* PASA provides a significant advance in the differentiation of micro-infiltration in early-stage colorectal cancer, highlighting its potential as an intraoperative diagnostic tool for pinpointing micro-infiltration.

## 2 Results

### 2.1 bUV-PAM for joint PAMI and *in situ* PASA

**Fig. 1:**
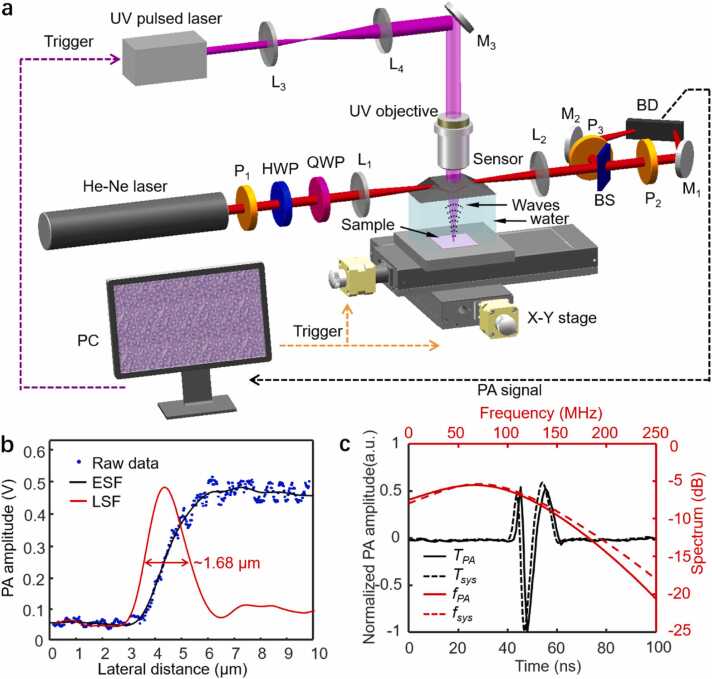
(a) Schematic of the bUV-PAM system incorporating the TIR sensor and the ultraviolet illumination laser for simultaneous PAMI and *in situ* PASA. P_1_-P_3_: polarizers; HWP: half-wave plate; QWP: quarter-wave plate; L_1_-L_4_: lenses; M_1_-M_3_: mirrors; BS: beam splitter; BD: balanced photoelectric detector; X-Y stage: two-dimensional electric scanning platform; UV: ultraviolet; PC: personal computer; PA: photoacoustic. (b) Lateral resolution of the bUV-PAM system. (c) Temporal profiles and their corresponding photoacoustic spectra of the measured photoacoustic signal and the system itself.

Fig. 1(a) illustrates the bUV-PAM system incorporating our previously proposed TIR sensor [28] for broadband photoacoustic detection and an ultraviolet illumination laser for nucleus-specific photoacoustic excitation. Specifically, the interrogating laser beam is manipulated through a polarizer, a half wave plate, and a quarter wave plate to produce tunable elliptical polarization, which is then applied to the prism-water interface of the TIR sensor to generate optical surface waves. A focused ultraviolet nanosecond pulsed laser excites the optically absorbing nuclei within the samples, generating broadband photoacoustic waves that perturb the water near the prism surface. The phases of the reflected polarized light beam are sensitive to the perturbation of the water's refractive index (n) caused by the ultrasonic pressure modulation. These phase shifts differ for the orthogonally p-polarized and s-polarized components, resulting in variations in the reflected light polarization status relative to its initial static condition. By incorporating a polarizer into the optical reflection path, the temporal fluctuations in the reflected light intensity can be accurately monitored, facilitating the measurement of the photoacoustic transients. For a comprehensive understanding of the system’s construction, refer to Section 3.2 of the Methods. The configuration of the bUV-PAM system allows for simultaneous PAMI and *in situ* PASA, aiming at creating a dual-modality virtual slice.

We determined the lateral resolution of the bUV-PAM by imaging a sharp blade edge using a scanning step size of 30 nm, as depicted in Fig. 1(b) [30], [31]. By analyzing photoacoustic amplitudes along the sharp edge, we derived the Edge Spread Function (ESF). Subsequently, the Line Spread Function (LSF) was obtained by taking the first derivative of the ESF, resulting in an estimated lateral resolution of approximately 1.68 μm, which is sufficient for imaging the nuclei of various tissues. The observed resolution slightly deviates from the theoretical diffraction-limited value calculated based on an objective with a Numerical Aperture (NA) of 0.13 and a laser wavelength of 266 nm. The deviation is primarily attributed to optical aberrations resulting from the excitation laser passing through media with different refractive indices, namely air, quartz, and water before reaching the sample.

We assessed the frequency response of the bUV-PAM system using an ultra-broadband photoacoustic pulse generated from a 100 nm graphene film. To protect the graphene film from potential ultraviolet laser-induced damage, we attenuated the photoacoustic excitation power to obtain an averaged photoacoustic signal. A time-resolved photoacoustic pulse (*T*_*PA*_) was captured by averaging 256 signals (shown as the black solid line in Fig. 1(c)) using a digital oscilloscope (MDO34, Tektronix) with a bandwidth of 1 GHz and a sampling rate of 5 GS/s. The Fourier-transformed power spectrum of this pulse (*f*_*PA*_) exhibited a remarkable −6 dB bandwidth of approximately 170 MHz, shown as the red solid line in Fig. 1(c). After conducting photoacoustic spectral calibration (detailed in Methods 3.3), we quantified the frequency response (*f*_*sys*_) of the bUV-PAM system using the formula *f*_*sys*_ = *f*_*PA*_ / *f*_*laser*_, determining an estimated −6 dB bandwidth of approximately 176 MHz, depicted as the red dashed line in Fig. 1(c). The corresponding time-resolved photoacoustic signal (*T*_*sys*_) is shown as the black dashed line in Fig. 1(c).

### 2.2 PAMI of tissue sections

**Fig. 2:**
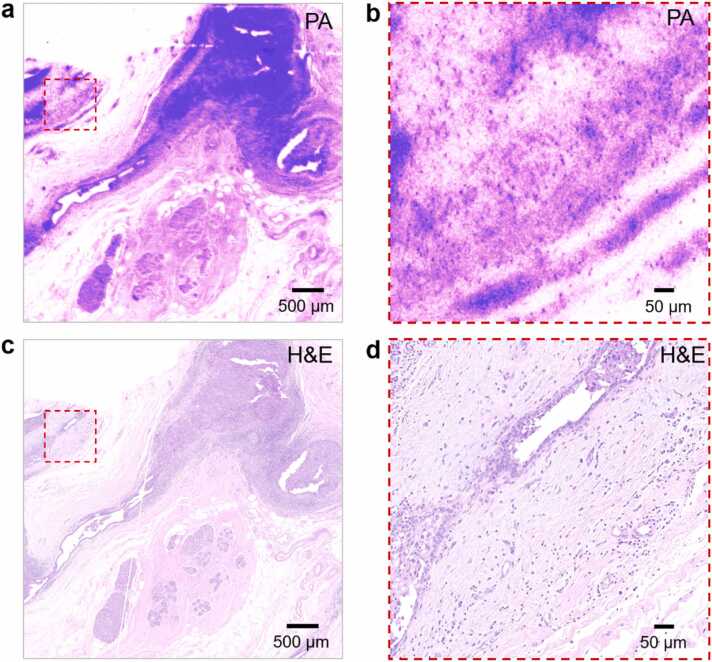
Comparison between the photoacoustic images and the H&E staining images of adjacent tissue sections. (a) Image of an unstained histopathological section acquired using the bUV-PAM system. (b) Magnified views of the regions marked by red dashed boxes in (a). (c) Standard H&E staining image of the tissue section adjacent to (a). (d) Magnified views of the regions marked by red dashed boxes in (c). PA: photoacoustic.

To substantiate the capacity of the bUV-PAM system to provide histological insights consistent with standard H&E staining, we conducted a comparative study between photoacoustic images of tissue sections and H&E staining images obtained with a conventional optical microscope [13]. Detailed methodologies for tissue preparation, label-free treatment, H&E staining, and photoacoustic imaging are delineated in Methods 3.4 and 3.5. We generated Maximum Amplitude Projection (MAP) images by extracting the peak photoacoustic amplitude from each A-line along the depth direction, employing false-color mode to augment visual contrast and facilitate comparison with H&E staining. Figs. 2(a) and 2(b) shows the photoacoustic MAP images of the histopathological section, while an adjacent section underwent H&E staining and was imaged under bright-field optical microscopy, depicted in Figs. 2(c) and 2(d). Compared to Figs. 2(a) and 2(c), the image obtained with the bUV-PAM system closely resembles the H&E staining image. In Fig. 2(b), the close-up photoacoustic images clearly exhibit well-defined nuclear distribution characteristics due to the specific ultraviolet absorption of the nuclei. The discrepancies observed between the close-up photoacoustic images (Fig. 2b) and their H&E counterparts (Fig. 2d) are mainly attributed to variations in tissue slices. These results indicate that bUV-PAM can capture label-free histopathological images closely matching H&E-staining images, underscoring its potential for the morphological pathological diagnosis of human tissues.

**Fig. 3:**
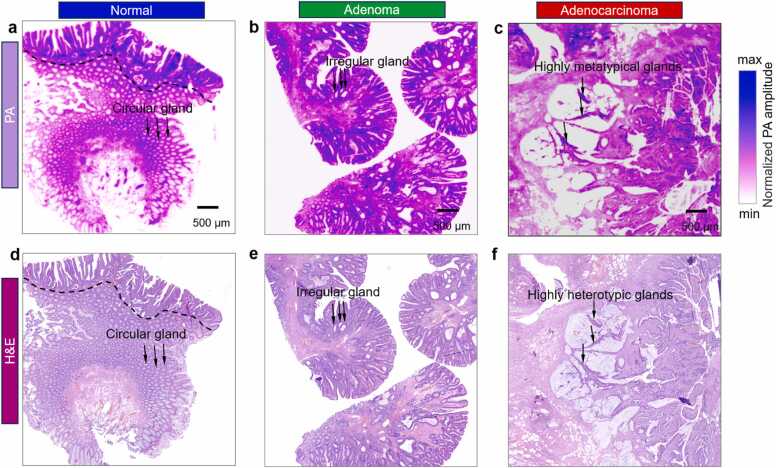
PAMI of human colorectal tissue sections. (a-c) Photoacoustic images of unstained normal, adenoma and adenocarcinoma colorectal tissue sections using the bUV-PAM system. (d-f) Standard H&E staining images of normal, adenoma and adenocarcinoma colorectal tissue sections adjacent to (a-c), respectively. Note that in (a) and (d), normal colorectal tissue is depicted below the black dash line. PA: photoacoustic.

Furthermore, to assess the morphological diagnostic capabilities of the photoacoustic system, we utilized the bUV-PAM system to capture images of unstained human colorectal tissue sections, including normal, adenoma, and adenocarcinoma samples, and compared them with H&E staining images (Fig. 3) [32], [33]. The photoacoustic image of normal colorectal tissue (beneath the black dash line in Fig. 3(a)) exhibits well-defined glandular arrangements with regular circular patterns, akin to its H&E staining counterpart (beneath the black dash line in Fig. 3(d)). Colorectal adenoma, a precancerous lesion characterized by atypical hyperplasia of glandular epithelial cells, manifests in the photoacoustic image (Fig. 3(b)) as denser and irregularly shaped glands, closely mimicking the H&E staining images in Fig. 3(e). The adenocarcinoma photoacoustic image (Fig. 3(c)) reveals remarkable pathological characteristics, such as highly metatypical glands, which closely match the corresponding H&E staining images (Fig. 3(f)). In summary, the bUV-PAM system adeptly captures essential histopathological diagnostic features within 20 min, facilitating label-free intraoperative diagnosis of normal, adenoma and adenocarcinoma colorectal tissue by pathologists.

### 2.3 PASA of human colorectal tissues

**Fig. 4:**
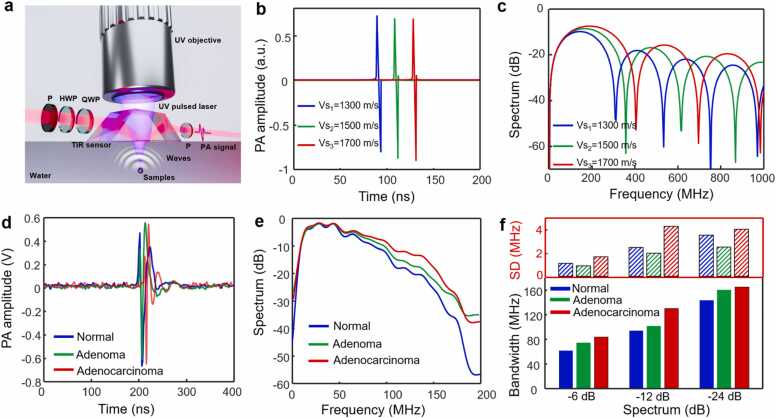
Theoretical and experimental research for diagnosing normal, adenoma and adenocarcinoma of colorectal tissues using the bUV-PAM system. (a) Schematic illustration of nuclear photoacoustic wave generation and detection using optical evanescent wave sensing. (b) Simulated time-domain photoacoustic signals and (c) the corresponding photoacoustic spectra representing different stages of colorectal tissue. Note that Vs_1_-Vs_3_ represent the acoustic velocity of nuclei covering different pathological states ranging from normal to adenocarcinoma. (d) Representative time-domain photoacoustic signals acquired from these colorectal tissues. (e) Experimental measurement averaged photoacoustic spectra curve of these colorectal tissues. (f) Statistical bandwidth characteristics of the photoacoustic spectral curves in (e). SD: standard deviation.

Schematic representation of nuclear photoacoustic wave generation and detection using optical evanescent wave sensor was shown in Fig. 4(a). Tissue nuclei with various pathological conditions can produce distinct photoacoustic spectrum curves when irradiated with an ultraviolet pulsed laser. The simulated details of the photoacoustic spectral generation are delineated in Methods 3.1. The theoretical time-domain photoacoustic signals from nuclei at different stages of colorectal tissue development, characterized by varying acoustic velocities, are shown in Fig. 4(b). Despite the similarity in signal profiles and amplitudes, which complicates the identification of disease progression stages, Fig. 4(c) reveals distinct photoacoustic spectral curves derived from the Fourier transform these time-resolved photoacoustic signals. These differences in spectral characteristics across different stages of colorectal tissues highlight the potential for differentiation between tissue types. Fortunately, by utilizing the broadband frequency response (approximately 176 MHz at −6 dB as depicted in Fig. 1(c)), our bUV-PAM system exhibits a capability for distinguishing between different types of colorectal disease. This capability underscores the potential utility of our system in the intraoperative assessment of colorectal pathologies, and offer a promising avenue for enhancing diagnostic accuracy during surgical procedures.

Further, we conducted an experimental PASA of human colorectal tissue samples at different stages of tumor progression, including normal, adenoma, and adenocarcinoma. Employing the photoacoustic spectral acquisition methodology detailed in Methods 3.6, we recorded photoacoustic signals from an ensemble of 10 samples and show 5 representative regions for each disease category in Figure S1. Fig. 4(d) shows typical time-domain photoacoustic signals for each tissue type, which underscore the challenge in distinguishing between disease states due to their analogous profiles and amplitude in the time domain. Upon performing a Fourier transform to all time-resolved photoacoustic signals and averaging their respective spectra (Fig. 4(e)). These spectra reveal discernible differences between the disease types, with adenocarcinoma exhibiting a broader bandwidth in the photoacoustic signal relative to adenoma and normal tissue. The quantitative metrics are presented in Fig. 4(f), where adenocarcinoma exhibits a bandwidth of approximately 84.0 MHz at −6 dB (standard deviation: 1.64 MHz), 130.8 MHz at −12 dB (standard deviation: 4.25 MHz), and 165.3 MHz at −24 dB (standard deviation: 4 MHz). Adenoma displays narrower bandwidths of about 75.0 MHz at −6 dB (standard deviation: 0.87 MHz), 101.8 MHz at −12 dB (standard deviation: 1.96 MHz), and 160.8 MHz at −24 dB (standard deviation: 2.47 MHz). Normal tissues exhibit the narrowest bandwidth, with approximately 62.0 MHz at −6 dB (standard deviation: 1.1 MHz), 94.25 MHz at −12 dB (standard deviation: 2.44 MHz), and 143.55 MHz at −24 dB (standard deviation: 3.5 MHz), respectively. The results affirm the effectiveness of PASA in differentiating among normal, adenoma, and adenocarcinoma colorectal tissues. Such findings are in accord with the theoretical expectations outlined in Fig. 4(c) and underscore the proficiency of the bUV-PAM system in discerning tissue types through the detection of broadband photoacoustic spectra, thus providing a consistent pathological assessment with H&E staining and photoacoustic morphological images.

The bUV-PAM system is capable of simultaneously diagnosing normal, adenoma, and adenocarcinoma colorectal tissue using PAMI (Fig. 3) or PASA (Fig. 4). The diagnostic results of these two methods are cross-verified, significantly improving the accuracy of pathological diagnosis. It's worth noting that the measured photoacoustic spectra might deviate from theoretical curves (Fig. 4(c)) due to the limited acoustic bandwidth (approximately 176 MHz at −6 dB) of the bUV-PAM system. Nevertheless, the bUV-PAM effectively distinguishes colorectal tissues across diverse developmental stages using PASA. We utilized conventional piezoelectric transducers with a center frequency of approximately 25 MHz to capture photoacoustic signals from tissues at different stages of colorectal disease. The time-resolved photoacoustic signals and their frequency spectra (Figure S2(a)) revealed consistent patterns across various colorectal diseases. Additionally, Figure S2(b) presents the photoacoustic signals and spectral data obtained using an ultrasound transducer with a center frequency of approximately 50 MHz, which exhibits similar contour characteristics but lacks the ability to discriminate between different stages of colorectal disease. The limitation arises from the fact that the distinguishing features in photoacoustic spectra typically occur within the high-frequency range of the acoustic spectral band (Fig. 4(c)). Consequently, detecting the subtle variations within the photoacoustic spectra of colorectal tissue becomes challenging when using a narrowband piezoelectric transducer.

### 2.4 Joint PAMI and PASA for micro-infiltrative assessment

**Fig. 5:**
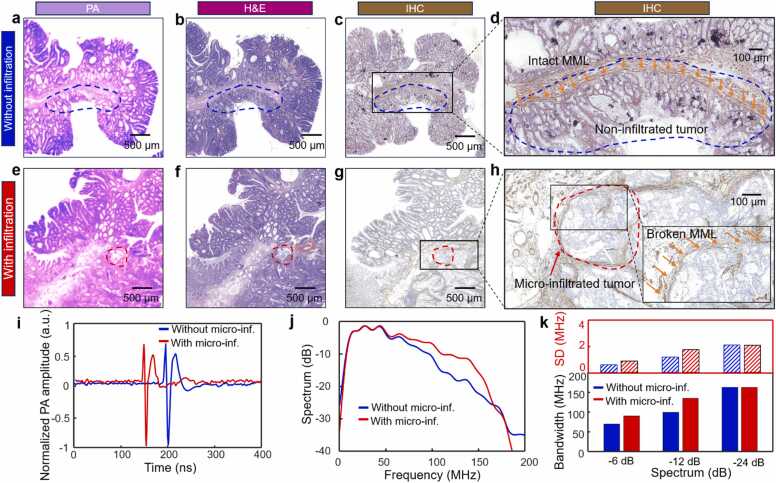
Assessment of micro-infiltration in colorectal adenoma HIN tissues using the joint PAMI and PASA approach. (a) and (e) Photoacoustic MAP images of colorectal tissues without and with micro-infiltration, respectively. (b) and (f) Standard H&E staining images of the colorectal tissues without and with micro-infiltration adjacent to (a) and (e), respectively. (c) and (g) Standard IHC biomarker SMA staining images of the colorectal tissues without and with micro-infiltration adjacent to (a) and (e), respectively. (d) and (h) Magnified views of the regions marked by black solid boxes in (c) and (g), respectively. (i) Representative time-domain photoacoustic signals acquired from the areas of interest of colorectal tissues in (a) and (e). (j) Experimental measurements of averaged photoacoustic spectra curves from the areas of interest in (a) and (e). (k) Statistical characteristics of photoacoustic spectral bandwidth corresponding to the curves in (j). Note that the regions of interest are enclosed by irregular shapes marked with blue or red dashed lines, in which the IHC images can identify the presence of early-stage cancer cells. PA: photoacoustic; micro-inf.: micro-infiltration; SD: standard deviation.

In colorectal adenomas, the progression from Low-grade Intraepithelial Neoplasia (LIN) to High-grade Intraepithelial Neoplasia (HIN) signals a significant risk of micro-infiltration, indicating of early-stage cancer [34], [35], [36], [37]. Figs. 5(a) and 5(e) present PAMI colorectal adenoma HIN tissue with or without micro-infiltration, respectively, acquired using the bUV-PAM. These images show highly morphological similarities to their corresponding H&E staining images in Figs. 5(b) and 5(f), which reveal a more densely packed glandular structure in comparison to the routine colorectal adenoma images in Figs. 3(b) and 3(e) [37]. Since the assessment of micro-infiltration in colorectal adenoma HIN using H&E or PAMI is challenging, pathologists typically confirm the presence of micro-infiltration by examining whether glandular epithelial cells have broken through the Mucosal Muscle Layer (MML). To facilitate this assessment, pathologists often resort to the time-consuming IHC technique using a biomarker, Smooth Muscle Actin (SMA), to better visualize the morphological characteristics of the MML [2], [3]. Figs. 5(c) and 5(g) display IHC images labeled with SMA, corresponding to the photoacoustic images (Figs. 5(a) and 5(e)) and the H&E staining images (Figs. 5(b) and 5(f)). It is important to note that the morphological characteristics depicted in IHC images are quite different from those in H&E staining or photoacoustic images. This disparity is mainly due to the fact that IHC focuses on molecular characteristics as opposed to the nuclear distribution highlighted in H&E staining or photoacoustic images.

Figs. 5(d) and 5(h) demonstrate a detailed view of the areas marked by the black solid line boxes in Figs. 5(c) and 5(g), respectively. In Fig. 5(d), the orange arrows highlight the intact MML, suggesting that the irregularly-shaped area enclosed by the blue dashed line is devoid of early cancerous cells. In contrast, Fig. 5(h) shows broken MML, indicated by the orange arrows, signifying the presence of early adenocarcinoma within the irregularly-shaped region bordered by red dashed lines. To facilitate the rapid assessment of early-stage colorectal cancer, we adopted the PASA method for distinguishing micro-infiltration by extracting and analyzing the characteristic photoacoustic spectra from diseased tissues using the bUV-PAM system. Fig. 5(i) depicts representative time-domain photoacoustic signals from the areas of interest delineated by the blue and red dashed lines in Figs. 5(a) and 5(e), respectively. The two curves show comparable characteristics in amplitude and contour. The corresponding averaged photoacoustic spectra of the regions are shown in Figs. 5(j) and 5(k), providing strong evidence that micro-infiltrated colorectal adenoma HIN tissues emit a photoacoustic signal with a broader bandwidth compared to those without micro-infiltration, in agreement with the theoretical analysis results in Fig. 4(c). Specifically, micro-infiltrated adenoma tissue emits photoacoustic signals with a bandwidth of approximately 91 MHz at −6 dB (standard deviation: 0.88 MHz), 136.3 MHz at −12 dB (standard deviation: 1.73 MHz), and 164 MHz at −24 dB (standard deviation: 2.07 MHz), while non-infiltrated adenomas exhibit bandwidths of around 70.3 MHz at −6 dB (standard deviation: 0.58 MHz), 99.8 MHz at −12 dB (standard deviation: 1.18 MHz), and 163.5 MHz at −24 dB (standard deviation: 2.09 MHz). These results demonstrate that the bUV-PAM system accurately distinguishes the micro-infiltration in colorectal adenoma HIN and consistently provides diagnostic results congruent with IHC (Figs. 5(d) and 5(h)), surpassing the diagnostic capacity of H&E staining images (Figs. 5(b) and 5(f)) or photoacoustic images (Figs. 5(a) and 5(e)). Consequently, the bUV-PAM holds potential as a valuable tool for intraoperative diagnosis of early-stage colorectal cancer.

To further validate the accuracy of photoacoustic spectral pathological diagnosis for micro-infiltration, we conducted PASA on additional 5 groups of non-infiltrated and micro-infiltrated samples. Figure S3(a_1_)-S3(e_1_) and S3(a_2_)-S3(e_2_) presents IHC staining SMA images of non-infiltrated and micro-infiltrated colorectal adenoma HIN tissues. In Figure S3(a_1_)-S3(e_1_), intact MMLs, denoted by orange arrows, indicated that the areas enclosed by the blue dashed line are devoid of early cancer. Conversely, in Figure S3(a_2_)-S3(e_2_), broken MMLs, denoted by orange arrows, indicates that the areas enclosed by the red dashed lines exhibit signs of early cancer. Next, we sampled time-domain photoacoustic signals from labeled regions corresponding to non-infiltrated and micro-infiltrated colorectal samples, extracting over 100 photoacoustic points in each region, and then transforming them into the frequency domain using Fourier transform. Figure S3(a_3_)-S3(e_3_) and S3(a_4_)-S3(e_4_) demonstrate that the average photoacoustic spectra and their corresponding statistical data effectively differentiate the mentioned disease types for multicase colorectal samples. Consequently, the bUV-PAM system has been validated for using the joint PAMI and PASA approach to distinguish micro-infiltration in colorectal adenoma HIN tissues.

## 3 Methods

### 3.1 Photoacoustic spectral simulation of human colorectal tissue with various developmental stages

To simulate the photoacoustic signal generation of the nucleus, we modeled the nucleus as a microsphere characterized by its radius (*a*) and acoustic velocity (*v*_*s*_). We calculated its photoacoustic spectrum using the following formula [24]:

(1) $$p_{f}\left(\hat{q}\right)=\frac{i\mu_{a}\beta I_{0}v_{s}a^{2}}{C_{p}r}\frac{[\left(\sin\hat{q}-\hat{q}\cos\hat{q}\right)]e^{-i\hat{q}\hat{\tau }}}{{\hat{q}}^{2}[\left(1-\hat{\rho }\right)\left(\sin\hat{q}/\hat{q}\right)-\cos\hat{q}+i\hat{\rho }\hat{c}\sin\hat{q}]}$$

Here, *β* represents the thermal expansion coefficient, *μ*_*a*_ is the optical absorption coefficient, and *C*_*p*_ is the specific heat capacity at equal pressure. The microsphere was heated by an optical beam with intensity *I*_*0*_ modulated at frequency *ω*. We defined two dimensionless variables, $\hat{q}$ and $\hat{\tau }$, which are frequency-dependent and time-dependent, respectively:

(2) $$\hat{q}=\frac{\omega a}{v_{s}}\mathrm{and}\hat{\tau }=\frac{v_{s}}{a}(t-\frac{r-a}{v_{f}})$$

In these equations, $\hat{\tau }$ represents the time delay from the edge of the microsphere, while the subscripts *s* and *f* correspond to the internal and external environments of the microspheres, respectively. In this study, the external environment is water. Additionally, we defined dimensionless variables $\hat{\rho }$ and $\hat{c}$:

(3) $$\hat{\rho }=\frac{\rho_{s}}{\rho_{f}}\mathrm{and}\hat{c}=\frac{v_{s}}{v_{f}}$$

Based on the theory that the photoacoustic spectral characteristics of the tissue are collectively influenced by factors, such as nucleus size, shape, density, and acoustic velocity, we explored the potential of these parameters in the diagnosis of early-stage adenocarcinoma. Morphological features of H&E staining images for the identification of micro-infiltration in colorectal tissues presents challenges. However, significant variations in the acoustic velocity of nuclei have been observed [25], [26], [27], and provide a promising route for accurate histopathological diagnosis. To model the progression from normal colorectal tissue to adenoma (including adenoma HIN without micro-infiltration) and then to adenocarcinoma (including adenoma HIN with micro-infiltration), we assigned acoustic velocity values of 1300 m/s, 1500 m/s, and 1700 m/s to the nuclei with human colorectal tissues, respectively. For accurate photoacoustic acquisition and to prevent nuclear overlap in the depth direction, we performed experiments on histopathological sections of approximately 6 μm thickness. We theoretically considered the nuclei of colorectal tissues as isotropic spheres with a diameter of 6 μm and an estimated density of 1000 kg/m^3^. A focused ultraviolet laser, which provides a lateral resolution of approximately 1.68 μm (Fig. 1(b)), was used for photoacoustic excitation, minimizing interference from the variations in nucleus size and shape.

### 3.2 Details of the bUV-PAM

The schematic of the bUV-PAM system is depicted in Fig. 1(a). In this work, a quartz prism, with refractive index of approximately 1.5 at 266 nm and 1.457 at 633 nm, supplied by Daheng Optics, was used to fabricate the TIR sensor. This prism is characterized by its exceptional light clarity, transmitting over 90 % of light with negligible interference from the 266 nm ultraviolet illumination laser. Designed with symmetrical base angles of approximately 29°, the prism facilitates precise beam path alignment, allowing incident light to closely approach the critical angle and reflect a parallel beam efficiently. The interrogation light, emitted by a continuous-wave He-Ne laser (HNL210LB, Thorlabs) with a wavelength of 632.8 nm and an output power of 21 mW, is polarized by a polarizer (P; LPVISE100-A, Thorlabs), a half-wave plate (HWP; WPH10M-633, Thorlabs), and a quarter-wave plate (QWP; WPQ10M-633, Thorlabs) to achieve tunable elliptical polarization. The beam was then focused onto the quartz-water interface through a lens (L_1_, with a focal length of 200 mm) initiating the generation of optical surface waves essential for acoustic detection. The laser-induced pressure transient changes the refractive index in the water, affecting the evanescent waves at the prism-water interface and causing a phase shift in the p-polarized and s-polarized components of the reflected light beam, while maintaining 100 % intensity reflectivity. After passing through a beam splitter with approximately 50 % transmission ratio (CCM1-BS013/M, Thorlabs Inc.), the split beams are further modified into time-varying linearly polarized beams by the incorporation of two orthogonal polarizers (P_2_ or P_3_; LPVISE100-A, Thorlabs). Subsequently, the beams are directed into two photodiodes within a balanced photoelectric detector (BD; PDB435a, Thorlabs) for differential detection, effectively suppressing background noise and enhancing the Signal-to-Noise Ratio (SNR). The photoacoustic signals are retrieved as temporal variations in light intensity difference between the beams. This signal output is further amplified using a low-noise amplifier (ZFL500NLþ, Mini-Circuits, Brooklyn, NY) and digitized by a data acquisition card (ATS9870, Alazar Technologies Inc., Pointe-Claire, QC, Canada; 450 MHz bandwidth; 1 GS/s sampling rate), facilitating high-fidelity signal retrieval.

For the excitation of nucleus-specific photoacoustic signals, we utilized an ultraviolet pulsed laser (Bright Solutions, Wedge HF 266) with a precise wavelength of 266 nm and a variable repetition rate of 1–40 kHz. The laser was collimated via a pair of lenses and subsequently directed onto the samples through an ultraviolet-transparent quartz prism to facilitate reflection-mode imaging. Focusing on the sample was achieved using an ultraviolet objective (Newport, U-13X-LC, NA = 0.13), ensuring precise alignment between the excitation laser and the acoustic detection unit for optimal photoacoustic responses. To enhance acoustic wave coupling, a thin water layer was introduced between the prism and the sample. Sample positioning and scanning were performed using a 2D scanning stage (L401–01, Shanghai JY Tech Co., Ltd), controlled by a custom LabVIEW script that managed laser emission, data acquisition, and raster scanning processes.

### 3.3 Photoacoustic spectral calibration

The spectral calibration process is essential for PASA to reduce detection interference and extract biologically relevant data [18]. In our study, we directed a pulsed laser onto an ultrathin graphene film (approximately 100 nm thickness), yielding a broadband, time-resolved photoacoustic signal. This signal was then subjected to Fourier transform to obtain the corresponding photoacoustic spectrum (*f*_*PA*_). The frequency response of the measured photoacoustic signal (*f*_*PA*_) is, in fact, the product of the bUV-PAM system's frequency response (*f*_*sys*_) and the original photoacoustic spectrum of the sample (*f*_*sam*_). Due to the acoustic transient time through the graphene film is significantly shorter than the laser pulse duration (approximately 1.2 ns at 25 kHz), the sample's photoacoustic spectrum (*f*_*sam*_) is nearly identical to the power spectrum of the ultraviolet illuminating laser (*f*_*laser*_). Therefore, the bUV-PAM system's frequency response (*f*_*sys*_) can be expressed as *f*_*sys*_ *= f*_*PA*_ */ f*_*laser*_. For the spectroscopic analysis of histopathological samples, we derived the autologous spectrum (*f*_*sam*_) using *f*_*sam*_ *= f*_*s*_ */ f*_*sys*_, considering their measured photoacoustic spectra (*f*_*s*_) and the pre-calibrated frequency response of the system (*f*_*sys*_).

### 3.4 Tissue section’s preparation

Clinical colorectal histopathological samples were obtained from the department of pathology and processed according to a standard protocol [38]. The samples, approximately 1.5 × 1.5 × 0.5 cm³ in size, were processed through formalin fixation preservation, gradient alcohol dehydration, and treatment with various organic reagents to enhance cellular structures. Subsequently, the samples were embedded in paraffin and sectioned into approximately 6 μm thick sections using a pathological microtome (RM2255, LEICA). These sections were then mounted on quartz sheets and baked at around 60 ℃ for 20 min. Due to the high optical absorption coefficient of paraffin at the wavelength of 266 nm, a thorough dewaxing process was necessary to eliminate unwanted photoacoustic signals. This involved heating the sections at 60 °C for one hour and immersing them in xylene for 20–30 min to remove all paraffin. The sections were then subjected to a series of ethanol washes (100 %, 90 %, 80 %, and 70 %), concluding with a final rinse in water to remove any residual xylene, thereby preparing the sections for subsequent photoacoustic imaging, H&E staining, and IHC staining. For H&E staining, the sections on quartz sheets were processed in a Leica automatic dye machine (ST5020, LEICA) equipped with hematoxylin and eosin dyes. This automated procedure produced staining sections for comparison with label-free photoacoustic images. For IHC staining, the sections were loaded into an IHC machine (Benchmark XT, ROCHE) for the biomarker SMA. This procedure aimed to assess the integrity of the MML and evaluate the potential presence of early-stage cancer in the tissue sample.

In this study, we employed paraffin sections for photoacoustic pathological diagnosis to obtain the superior detail of H&E staining compared to frozen sections [40]. This method enables pathologists to more accurately diagnose the type of diseases and location of affected tissue when correlating with photoacoustic findings. In addition, paraffin sections are irreplaceable by frozen sections for the IHC biomarker SMA, which is critical for identifying micro-infiltration in colorectal adenoma HIN. The discrepancy in characteristics between PAMI and PASA across various diseases primarily attribute to the internal structural and molecular properties of the colorectal tissue. Thus, it is suggested that diagnostic results from photoacoustic techniques should be consistent between frozen and paraffin sections. Consequently, the intraoperative frozen sections appear to be a viable option for photoacoustic histopathological assessment. [39], [40]

### 3.5 Photoacoustic image acquisition

Photoacoustic imaging of colorectal tissues was conducted using an illumination laser with a Pulse Repetition Frequency (PRF) of 25 kHz and a scanning step size of approximately 1.0 μm. The photoacoustic imaging technique demonstrated the ability to capture a volumetric image with dimensions of 5000 pixels (*x*) × 5000 pixels (*y*) × 64 pixels (*z*), covering a Field of View (FOV) of 5 × 5 mm^2^, within approximately 20 min. Each time-resolved photoacoustic A-line signal generated by the laser pulse was recorded and digitized using a data acquisition card. Since the thickness of samples was smaller than the depth resolution of the ultraviolet bUV-PAM system, we reconstructed the photoacoustic images by performing a MAP of each A-line signal along the depth direction, as shown in Figs. 2(a) and (b), 3(a)-3(c), 5(a) and 5(e).

### 3.6 Photoacoustic spectral acquisition

Before acquiring photoacoustic spectra, it is crucial to accurately diagnose disease types and delineate the boundaries of different colorectal tissues to ensure each spectral curve corresponds to its respective histopathological sample. To achieve this, we employed classical H&E staining for precise disease classification, enabling normal, adenoma, and adenocarcinoma regions to be labelled within unstained colorectal tissues by exploiting the similarities between photoacoustic and H&E images. Since H&E images are inadequate for evaluating the micro-infiltration of colorectal adenoma HIN tissues, we employed the commonly used IHC biomarker SMA images to effectively classify and label relevant regions. To obtain photoacoustic signals corresponding to specific disease types, we utilized a two-dimensional electric scanning stage to position the sample at particular areas. We randomly collected approximately 100 photoacoustic signals in each of 10 samples for normal, adenoma, and adenocarcinoma colorectal tissues, and in each of 6 samples for adenoma HIN with or without micro-infiltration, respectively. To minimize potential laser radiation damage to tissue samples, we employed a significantly reduced energy level of 50 nJ for the photoacoustic excitation and used an average mode acquisition of 128 times to improve the SNR of the photoacoustic signal, thereby reducing noise interference and enhancing the accuracy of PASA. Subsequently, the time-resolved photoacoustic signals were Fourier transformed to extract their respective photoacoustic spectra. After averaging all the photoacoustic spectra of each disease type, we obtained representative photoacoustic spectral curves corresponding to specific disease types. Using the formula *f*_*sam*_=*f*_*s*_ / *f*_*sys*_ for photoacoustic spectral calibration, we retrieved the original spectra of colorectal tissues from different disease types, allowing us to derive the photoacoustic spectra of the measured tissues themselves.

## 4 Discussion

During surgical procedures, accurately assessing the pathological nature of surgical margin is critical to ensure complete excision of malignant cells, reduce the risk of disease recurrence and enhance surgical outcomes [41], [42], [43]. Current intraoperative pathological diagnosis methods primarily rely on evaluating morphological characteristics in H&E staining images of frozen sections to determine the status of surgical margin. However, accurately evaluating micro-infiltration through H&E staining images is challenging, making it difficult to identify early-stage cancer signs within the surgical margin. Consequently, this can lead to overly wide surgical margins or incomplete tumor resections, significantly impairing overall surgical efficacy. Although the ability of IHC to distinguish micro-infiltration in colorectal adenoma is noteworthy, its clinical application during intraoperative procedures is constrained by the prolonged duration required for biomarker assessment.

In this paper, we present a novel bUV-PAM that enables joint PAMI and *in situ* PASA for rapid intraoperative assessment of micro-infiltration within 20 min (Fig. 1). Our approach enables label-free, precise morphological imaging of normal, adenoma, and adenocarcinoma colorectal tissues, which is consistent with traditional H&E staining results (Fig. 3), and distinguishes these tissue types by detecting significant photoacoustic spectral changes (Fig. 4), thereby validating the feasibility of the joint PAMI and in suit PASA method. Significantly, this approach effectively identifies micro-infiltration in colorectal adenomas HIN, offering a valuable tool for enhancing the intraoperative assessment of surgical margins and thereby improving surgical outcomes (Fig. 5). This advancement not only establishes an intraoperative diagnostic method for early-stage colorectal cancer but also shows promise for diagnosing other micro-infiltrated cancers, such as breast Ductal Carcinoma in Situ (DCIS) [44], [45] and Squamous Cell Carcinoma in Situ (SCCIS) [46], [47], which are traditionally difficult to diagnose using H&E staining or IHC methods.

The bUV-PAM system encounters challenges in clearly visualizing the morphological features of individual nuclei due to the limited sensitivity of the optical evanescent wave sensor and it’s lateral resolution. Advanced sensing techniques, such as plasmonic waveguide resonance [48] and plasmonic metamaterials [49], significantly improve the SNR of photoacoustic imaging while preserving a broad bandwidth. Achieving higher lateral resolution requires a shorter step size cooperating with high NA ultraviolet objectives while maintaining the same imaging time, requiring a higher repetition rate ultraviolet laser and innovative scanning technologies featuring a large FOV and high scanning speed [50]. By addressing these challenges, our bUV-PAM system could capture the histology visualizations that are comparable to the H&E staining images. Combined with deep learning virtual staining techniques [13], [51], [52], [53], this system may enable the reconstruction of virtual staining photoacoustic images that closely resembling to H&E staining. Wavefront engineering of ultraviolet light field provides a large depth of field [54], [55], extending focal depth with subcellular lateral resolution within an axial enlarged depth region. By integrating our bUV-PAM system that possesses an excellent axial resolution of ∼7.5 μm [28], the photoacoustic histology imaging could be obtained in fresh samples, providing the tomographic images akin to H&E images from the mechanically sectioned slices.

The accuracy of artificial intelligence in predicting IHC results primarily depends on the direct correlation between the original and the IHC images. Traditional methods primarily use H&E staining images as data source that mainly highlight nuclear size, morphology, and distribution, but fail to reveal correlations with the protein expression in IHC images. [6], [7] Additionally, as the H&E and IHC images are always derived from different sections, many limitations, such as image distortion, folding, and rotation during preparation, pose significant challenges for achieving pixel-level image registration. In contrast, our bUV-PAM system can acquire label-free morphological images comparable to the H&E images, while simultaneously capturing photoacoustic spectral information that directly correlates with IHC results [56]. Furthermore, our photoacoustic technology obviates staining operation, allowing both photoacoustic data acquisition and IHC biomarker on the same sections, thus obviously simplifying image registration. Consequently, the joint PAMI and PASA technologies is expected to improve the accuracy of IHC results predictions.

In summary, the bUV-PAM system, featuring broadband frequency response and targeted ultraviolet photoacoustic excitation, effectively conducts joint PAMI and *in situ* PASA on various human colorectal tissue pathological samples, accurately distinguishing micro-infiltration. We believe that the bUV-PAM system has significant practical value for intraoperative tumor diagnosis, particularly in early-stage cancer assessment.

## Ethics approval and consent to participate

The present study was approved by the ethical review committee of Tianjin Union Medical Center of Nankai University. Written informed consent was obtained from all enrolled patients.

## Funding sources

This work was supported in part by Guangdong Major Project of Basic Research: 2020B0301030009; National Natural Science Foundation of China (NSFC): 62405289, 62175159, 12074203, 12174204; National Key Research and Development Plan of China: 2023YFF0715300; 10.13039/501100003453Natural Science Foundation of Guangdong Province: 2023A1515012888; The Science and Technology Innovation Commission of Shenzhen: JCYJ20220818101417039, JCYJ20241202124428038; 10.13039/501100012234Shenzhen Peacock Plan: KQTD20170330110444030; Medical-Engineering Interdisciplinary Research Foundation of Shenzhen University: 86901/00000311; Scientific Instrument Developing Project of Shenzhen University: 2023YQ001; Shenzhen University 2035 Initiative: 2023B004; Key R&D Program of Zhejiang: 3000-3AA240100.

## Author contributions

Wei Song, Yong Yang and Xiaocong Yuan conceived and designed the study; Fan Yang and Zhengduo Yang performed the experiments; Zhengduo Yang and Siwei Zhu performed bioinformatics analysis; Fan Yang, and Zheng Zhu collected and analyzed the data. Fan Yang, Wei Song, Yong Yang and Xiaocong Yuan interpreted the results and wrote the manuscript. All authors read and approved the final manuscript.

## Consent for publication

Patients agreed to participate in this work.

## Associated Content

Supporting Information is available for this paper.

## CRediT authorship contribution statement

**Yang Zhengduo:** Writing – review & editing, Validation, Methodology, Investigation, Conceptualization. **Yang Fan:** Writing – review & editing, Writing – original draft, Visualization, Validation, Software, Investigation. **Yang Yong:** Writing – review & editing, Supervision, Formal analysis. **Song Wei:** Writing – review & editing, Resources, Methodology, Conceptualization. **Zhu Siwei:** Resources, Project administration, Funding acquisition. **Zhu Zheng:** Software, Investigation. **Yuan Xiaocong:** Writing – review & editing, Resources, Project administration, Methodology, Conceptualization.

## Declaration of Competing Interest

The authors declare no competing financial interests.

## Data Availability

No data was used for the research described in the article.
