# Supplementary material for: A joint photoacoustic imaging and broadband spectral analysis for early-stage intraoperative pathology assessment: A case study with colorectal cancer
Source: Photoacoustics. 2025 Mar 4;43:100712. doi: 10.1016/j.pacs.2025.100712 (PMC11929096; doi:10.1016/j.pacs.2025.100712)
Supplement: Supplementary file 1 — Supplementary material [file mmc1.docx]

**Supplementary materials for**

**A joint photoacoustic imaging and broadband spectral analysis for early-stage intraoperative pathology assessment: a case study with colorectal cancer**

Fan Yang^1,2,^^#^, Zhengduo Yang^3,#^, Zheng Zhu^1^, Siwei Zhu^4^, Wei Song^2,🖂^, Yong Yang^1,🖂^, Xiaocong Yuan^1,2,🖂^

1 Research Center for Frontier Fundamental Studies, Zhejiang Laboratory, Hangzhou, 311100, China

2 Nanophotonics Research Center, Shenzhen Key Laboratory of Micro-Scale Optical Information Technology, Institute of Microscale Optoelectronics, Shenzhen University, Shenzhen, 518060, China

3 Department of Pathology, Tianjin Union Medical Center, Tianjin, 300121, China

4 The Institute of Translational Medicine, Tianjin Union Medical Center of Nankai University, Tianjin, 300121, China

# These authors contributed equally.

🖂 Corresponding authors: Wei Song ([weisong@szu.edu.cn](mailto:weisong@szu.edu.cn)); Yong Yang (yangyong@zhejianglab.edu.cn); Xiaocong Yuan(xcyuan@szu.edu.cn).

**S1 The standardized H&E staining images of colorectal tissue sections depicting normal, adenoma, and adenocarcinoma specimens**


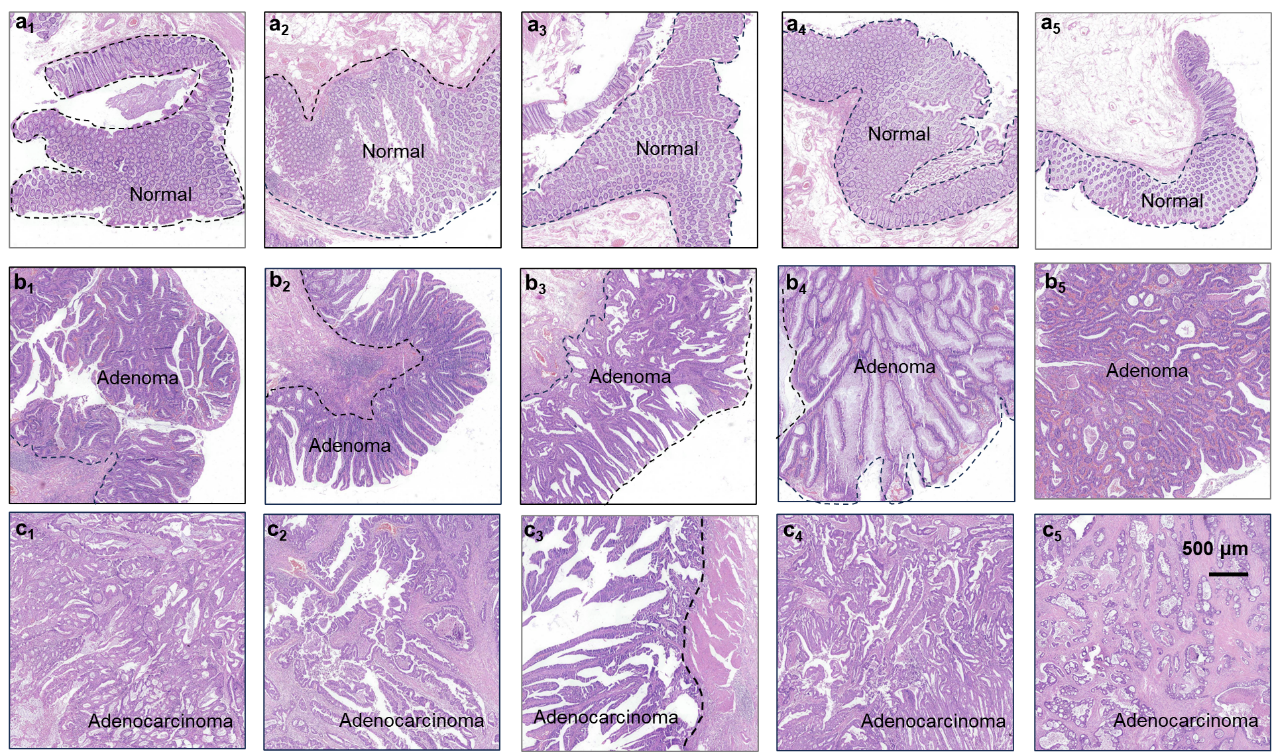


Figure S1. The H&E staining images of colorectal pathological sections representing normal, adenoma, and adenocarcinoma. (a_1_-a_5_) H&E staining images of the normal colorectal tissue sections. (b_1_-b_5_) H&E staining images of the colorectal adenoma sections. (c_1_-c_5_) H&E staining images of the colorectal adenocarcinoma sections.

Figure S1 shows the H&E staining images of standard colorectal tissue sections, depicting normal, adenoma, and adenocarcinoma development stages, each stage consisting of 5 cases, which served as the good standard images for precise disease classification, facilitating accurate the photoacoustic spectral acquisition across different stages of disease progression.

**S2 Photoacoustic spectrum assessment of human normal, adenoma and adenocarcinoma colorectal tissues acquired using transducers**

Figure S2 shows the time-domain photoacoustic signals and their power spectra of normal, adenoma and adenocarcinoma colorectal tissues acquired using piezoelectric transducers with center frequency of approximately 25 MHz and 50 MHz, respectively. These photoacoustic spectral curves display analogous contour features, failing to distinctly differentiate between normal, adenoma, and adenocarcinoma colorectal tissue.


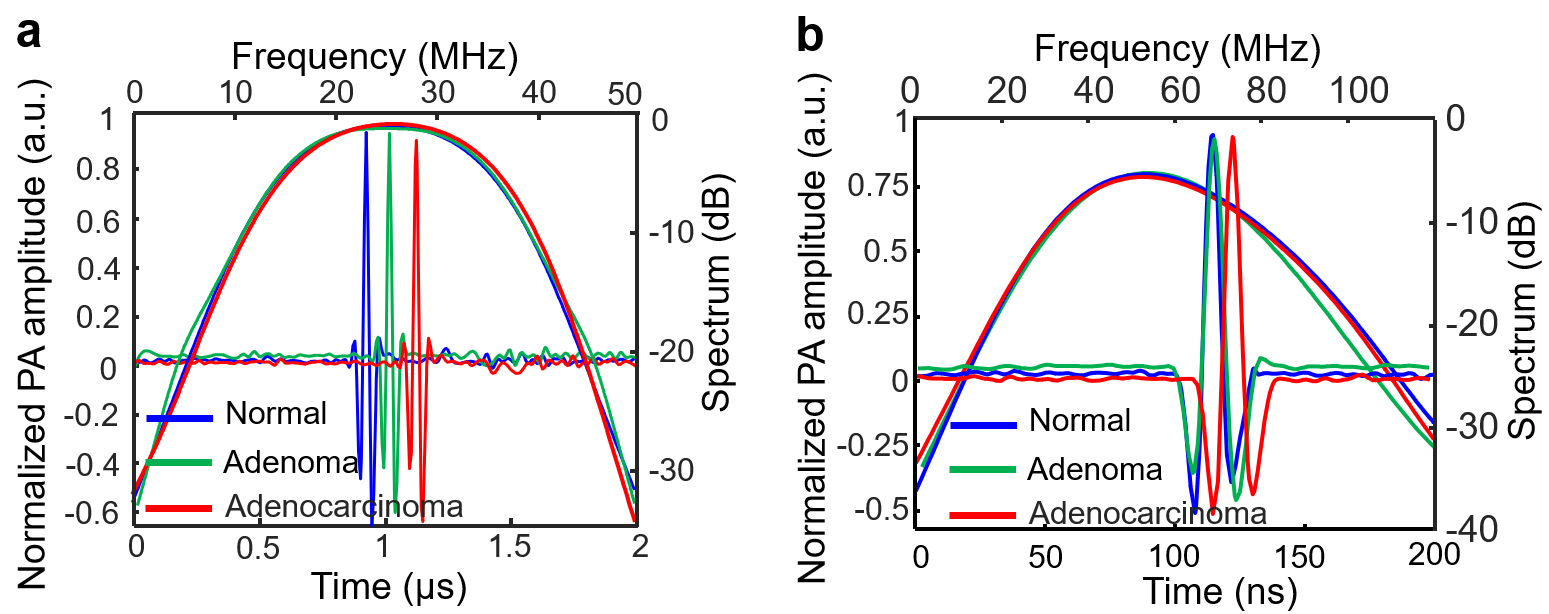


Figure S2. Photoacoustic spectral assessment of human colorectal tissues using transducers. (a) Time-domain photoacoustic signals and their power spectra of human normal, adenoma and adenocarcinoma colorectal tissues acquired using piezoelectric transducers with a center frequency of approximately 25 MHz. (b) Time-domain photoacoustic signals and their power spectra of human normal, adenoma and adenocarcinoma colorectal tissues acquired using piezoelectric transducers with a center frequency of approximately 50 MHz. PA: photoacoustic.

**S3 IHC staining SMA images and their photoacoustic spectra of human colorectal tissues without and with micro-infiltration**

Figure S3(a_1_)-S3(e_1_) and S3(a_2_)-S3(e_2_) present 5 groups of IHC staining SMA images of colorectal tissue sections, illustrating regions of colorectal adenoma HIN with and without micro-infiltration. Figure S3(a_3_)-S3(e_3_) and S3(a_4_)-S3(e_4_) depict photoacoustic spectral curves and their statistical bandwidth characteristics for the corresponding tissues in Figure S3(a_1_)-S3(e_1_) and S3(a_2_)-S3(e_2_), respectively. These outcomes align with the findings in Figure 5, providing compelling evidence for the accurate differentiation of micro-infiltrations in human colorectal adenoma HIN tissue through the use of the broadband PASA method.


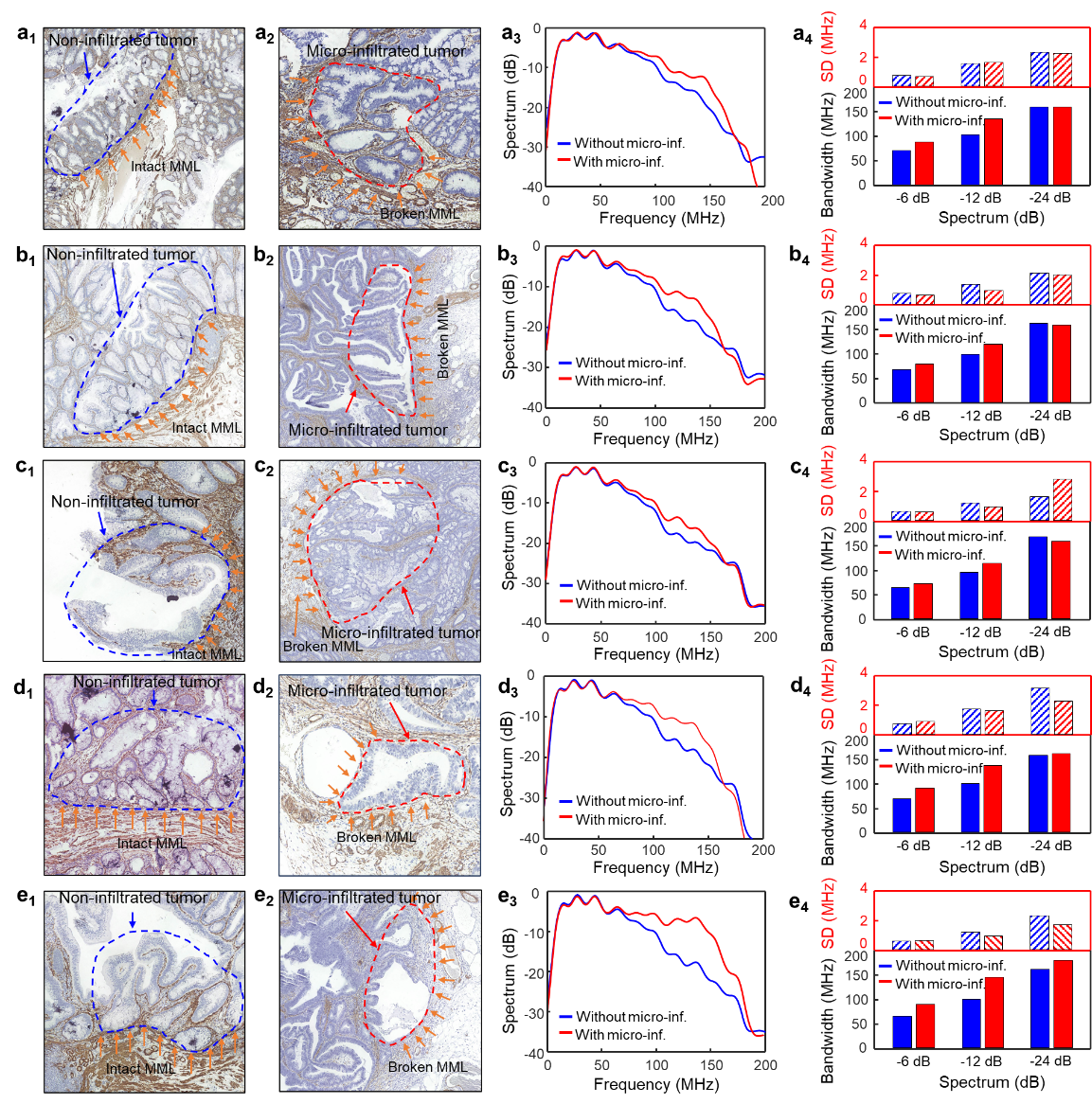


Figure S3. IHC staining SMA images and their corresponding photoacoustic spectra of human colorectal adenoma tissues with and without micro-infiltrations. (a_1_)-(e_1_) IHC images demonstrating non-infiltrated colorectal tissues, where the MML, indicated by orange arrows, remains intact, suggesting that the nuclei within these blue dash lines do not indicate early cancer. (a_2_)-(e_2_) IHC images displaying micro-infiltrated colorectal tissues with broken MML, as pointed by orange arrows, indicating that the nuclei within these red dash lines represent early-stage cancer. (a_3_)-(e_3_) Experimentally measured averaged photoacoustic spectra curves of the colorectal tissues in (a_1_)-(e_1_) and (a_2_)-(e_2_), respectively. (a_4_)-(e_4_) Statistical characteristics of the photoacoustic spectral bandwidth observed in the curves of (a_3_)-(e_3_), respectively. micro-inf.: micro-infiltration; SD: standard deviation.
